# Nonlinear modeling of venous leg ulcer healing rates

**DOI:** 10.1186/1471-5945-9-2

**Published:** 2009-03-31

**Authors:** Matthew Cardinal, Tania Phillips, David E Eisenbud, Keith Harding, Jonathan Mansbridge, David G Armstrong

**Affiliations:** 1 Advanced BioHealing, La Jolla, CA, USA; 2 Boston University School of Medicine, Boston, MA, USA; 3 Southern Arizona Limb Salvage Alliance (SALSA), Department of Surgery, University of Arizona, Tucson, Arizona USA; 4 Cardiff University School of Medicine, Wales, UK; 5 Tecellact, La Jolla, California, USA

## Abstract

**Background:**

The purpose of this manuscript was to determine whether the change in wound surface area over time could be described through nonlinear mathematics.

**Methods:**

We studied 3,588 serial wound tracings of 338 venous leg ulcers (VLUs) that had been followed during a controlled, prospective, randomized trial of two topical wound treatments.

**Results:**

A majority (72%) of VLUs exhibited surface area reduction via an exponential decay model, particularly during the early stages of healing. These results were consistent with the mechanics of wound contraction and epithelial cell proliferation, supported by the higher frequency at which exponential surface area reduction associated with full wound closure (35% of wounds that fit the exponential model healed vs. 21% of wounds that did not fit the exponential model completely healed during the study period, p = 0.018). Goodness-of-fit statistics suggested that much of the individual variation in healing could be described as nonlinear variation from the exponential model.

**Conclusion:**

We believe that parameter estimates from a mathematical model may provide a more accurate quantification of wound healing rates, and that similar models may someday reach routine use in comparing the efficacy of various treatments in routine practice and in product registration trials.

## Background

While sophisticated mathematical analyses have been used to model the kinetics of wound closure, such models have not reached widespread acceptance among clinicians or regulatory authorities. Healing rates continue to be most widely reported as percent surface area reduction over time, and registration trials typically use a binomial outcome (i.e. completely healed vs. not completely healed on a chosen day) to judge product efficacy. Many clinicians realize that such an approach is problematic, and that such analysis fails to describe adequately to what extent a wound has healed in response to its treatment regimen. In addition, a reliable model that would enable one to predict chance of healing and to project the time to complete healing would be useful in the clinical arena. Quantitating intermediate degrees of healing is critical in order to decide whether treatment is successful and to make therapeutic decisions at each patient visit.

The field of wound healing is rapidly expanding. This rapid expansion, we believe, is happening at a rate that is surpassing the development of adequate operational definitions of success and therapeutic progress. It would be ideal to have a mathematical model for wound healing kinetics, expressed in physiological terms, which could provide a surrogate marker for prognostication. Such a model could enhance a clinician's ability for timely therapeutic decisions and it may shorten the duration of product registration trials. Enabling the patient to have realistic expectations of healing time also would lead to higher satisfaction and planning for vacation and return to work. In this study we have assessed the applicability of least squares curve fitting using generalized exponential equations to derive the healing rates of serially measured VLU tracings, and have used a large database of serial wound observations to test this model.

## Methods

The wound surface area data used in the present study was derived from the trial of Dermagraft^® ^for treating VLU, which was completed in 2004. This trial was not listed with the registries that have become commonplace in subsequent years. The clinical protocol for the trial was reviewed and approved by the appropriate Institutional Review Boards (IRB). Patients were required to sign IRB-approved Informed Consent forms before randomization to treatment. In this investigation 366 patients with chronic venous ulceration finished a brief run-in period of standard compression therapy and then were randomized to receive Dermagraft^® ^plus compression, or compression therapy alone. Patients were followed weekly until they were healed or until the 12-week active treatment phase was complete. All patients were followed to week 24 to assess wound status post-treatment. The primary efficacy endpoint of the trial was 100% ulcer closure (re-epithelialization) by week 12 of the treatment phase.

All wound tracings were subjected to computerized planimetry measurements. Only patients that attended enough study visits (4) to obtain sufficient trace data for nonlinear regression analysis were included in this study. Data from 338 wounds were found to include a minimum of 4 separate observations of computerized planimetry derived surface area. Each wound measurement (cm^2^) was assigned as a function output *y *such that the data could be fitted to a generalized exponential equation:

*y *= a * *e*^*bx*^

where *a *and *b *are the parameter estimates for initial wound size (cm^2^) and healing rate (week^-1^), respectively, and *x *represents the corresponding time (weeks) of each wound measurement. The algorithm returned the estimated values for wound size, healing rate, 95% confidence intervals of the parameter estimates, as well as standard goodness-of-fit regression statistics (e.g. coefficient of determination r^2^) for each regression fitting.

Curve fitting algorithms were processed using MATLAB 7.4.0 Curve Fitting Toolbox (The Mathworks Inc., Natick, MA). Correlation coefficient critical values using an adjusted-r^2 ^were determined at a significance requirement of α = .05. Summary statistics and hypothesis tests such as Mann-Whitney, ANOVA, and T-tests on model coefficients were executed using MATLAB Statistics Toolbox and Minitab 15.1 (Minitab Inc., State College, PA). All tests for significance were 2-tailed at α = .05 Only data collected during the 12-week active treatment phase were included because we believed that including the discontinuous point at 24 weeks, without intervening observations during weeks 13–23, would skew the model; i.e. all patients who healed during this interval would be considered healed at 24 weeks.

## Results

**Figure 1:**
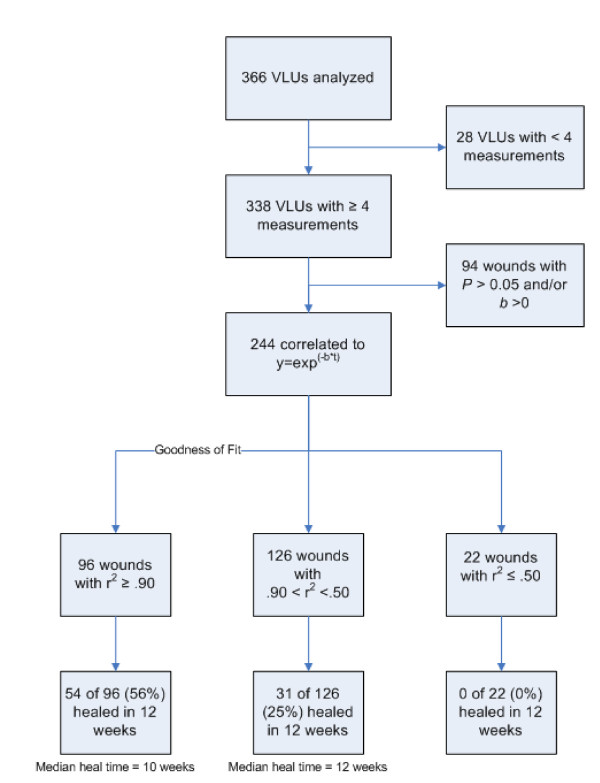
**Flow chart of clinical and regression analysis results**.

**Figure 2:**
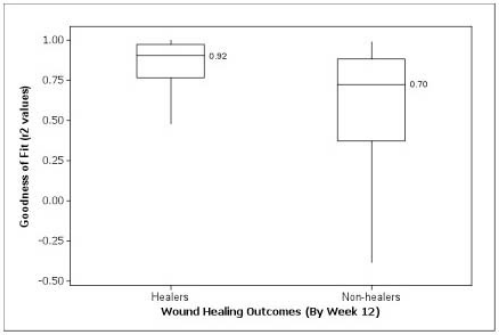
**Boxplot (with medians) of goodness-of-fit statistics (r^2^) between VLU patients who healed and did not heal by Week 12**.

Many of the wound curves analyzed exhibited patterns of exponential change in wound size. 244 out of 338 wounds (72%) achieved correlation to an exponential decay (*e*^-*bx*^) model, as judged by statistical significance (p < .05), with a median adjusted-r^2 ^= 0.87. Of the 105 VLUs that were clinically assessed as healed during the 12-week treatment phase of the trial, 85 (81%) met significant correlation requirements to the exponential decay model, with a median r^2 ^= 0.92 (Figures 1, 2). The value of the coefficient of determination r^2 ^represents how well each wound curve fit an exponential decay curve. Thus, for half of the 105 VLUs that did heal during treatment, we can say that 92% or more of the variation in wound size can be explained as an exponential decrease in size.

The estimated healing rates (*b*) for the significant exponential decay fits were much larger than those for the wound curves not following this pattern, with a median healing rate 29.5 times larger (-.177 weeks^-1 ^vs. -.006 weeks^-1^, p < .001), suggesting that regression significance was associated with wounds that were healing. Wound curves that correlated significantly to exponential decay were also more frequently associated with wounds that completely re-epithelialized than wound curves that did not reach statistical correlation or correlated to an exponential growth curve (35% vs. 21%, p = 0.018). The regression model most often failed to match observed healing patterns in VLUs for wounds that did not correlate to exponential decay or matched patterns of growth in size (94 of 338, 28%).

**Figure 3:**
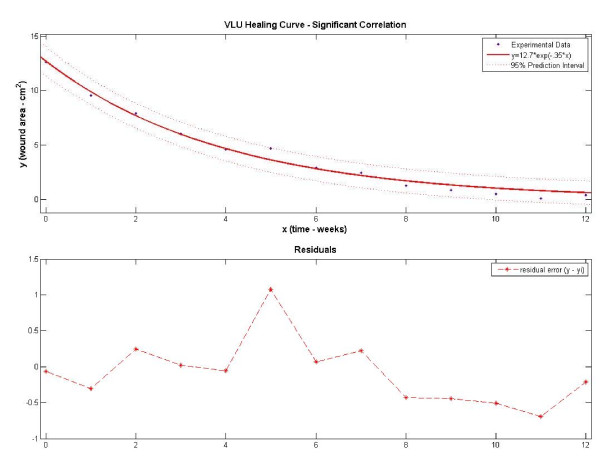
**Experimental and fitted regression data using a significantly correlated VLU data set**. Residuals are distributed randomly about zero and are generally small in magnitude.

**Figure 4:**
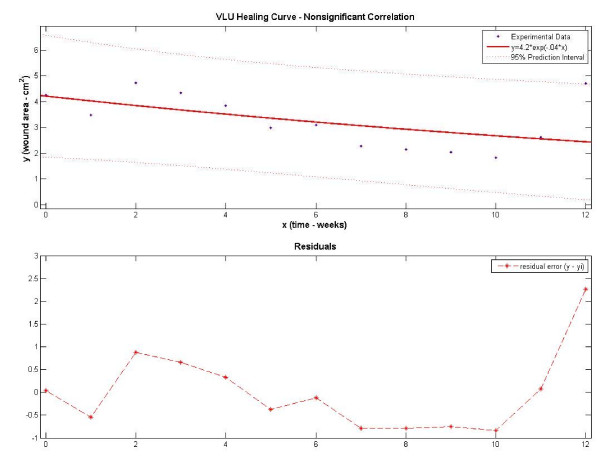
**Experimental and fitted regression data using a nonsignificantly correlated VLU data set**. Residuals are distributed less randomly about zero and **are larger in **magnitude, particularly at week #12 (2.27). Unusually large residuals are typically not attributable to regression variation, resulting in a poor model fit.

Examples of significantly and nonsignificantly correlated wound curves are displayed in Figures 3 and 4, respectively. In Figure 3 the VLU wound surface reduction follows an exponential decay pattern with a healing rate constant of -0.25 weeks^-1^. The residual variation, a measure of the difference between the observed wound size and the predicted size, is small and the result is an adjusted-r^2 ^of 0.98 (p < .001). Due to the high goodness-of-fit, we can predict that the surface area of this VLU will continue to reduce and approximate to 0.49 cm^2 ^(0.0 – 1.4 cm^2^, 95% CI) in week 13. In Figure 4 the wound surface area is slowly reducing with an estimated healing rate constant of -0.04 weeks^-1^. The observed wound size varies from the regression prediction, particularly at week 12 (residual 2.27 cm^2^) where the prior wound size reduction is reversed by a large spike in wound size. The result is a poor fit to an exponential decay equation (adjusted-r^2 ^= 0.20, p = .066). Nonsignificant statistical correlations (p-values > .05) and/or weak fits (r^2 ^< .50) to the model may compromise the use of estimated healing rates to predict future wound sizes.

## Discussion

Biological events rarely occur with linear kinetics and attempting to describe wound repair with any single simple formula is problematic, since the healing process is a complex combination of events such as re-epithelialization and contraction. Further, wounds present in a wide range of sizes, and "50 percent healing" of a large wound represents much more extensive cellular proliferation and migration than a similar degree of linear healing in a tiny lesion.

Several studies have shown that wound contraction can be described by an exponential curve. In a classic study on full-thickness rabbit wounds, the reduction in the logarithm of wound area over time corresponded to a series of linear vectors, indicating a logarithmic process of contraction [1]. In a subsequent investigation, the closure of excised wounds in rats was shown to follow an exponential decrease in wound area during the contraction phase, supporting the notion of a contraction decay constant could describe the rate of wound closure, independent of lesion size and geometry [2]. Porcine full thickness wounds have been shown to correlate to an exponential decay of area over time [3,4]. A mechanistic wound contraction model confirmed the exponential relationship of wound contraction versus time, and the size-and-shape independence of the contraction decay constant [5]. Numerical solutions of a wound contraction mathematical model yielded comparable results to the excised rat wounds [6]. An analysis of contraction inhibition in hairless mice also indicated an exponential decrease in wound area during the first half of the study period [7].

Research on epithelialization and keratinocyte proliferation models has produced evidence of both linear and nonlinear patterns of wound healing. One might expect, a priori, that the exponential nature of cell division might lead to ever accelerating epithelial growth and wound closure. However, both cell-to-cell contact inhibition and the limited ability of keratinocytes to divide before they reach senescence limit this acceleration and prevent theoretical prediction about the kinetics of re-epithelialization. Further, the keratinocytes on the healing edge of a wound are restricted to proliferation and migration towards the open wound area, in contrast to the centrifugal growth of a cell colony observed in vitro. Models based on the relationship of cell proliferation to the rate of increase in cell density and chemical concentration produced numerical solutions exhibiting a lag and linear phase in reduction of wound radius over time [8]. Cell fronts in assays have been shown to travel with constant speeds under assumption of a logistic cell proliferation rate [9]. Another research group assumed an exponential growth pattern for the proliferation rate parameter of clonal subtypes in a study on aerosolized skin graft modeling [10]. Elsewhere, a delayed exponential model was used to predict time to full re-epithelialization in chronic wounds treated with electrical stimulation [11]. Exponential patterns of epithelialization have also been observed in corneal defects [12]. A significant limitation of in vitro and animal studies is that they typically study the response to a one-time injury rather than mimicking the repeated damaging effects of venous hypertension, matrix metalloproteinases, trauma, etc. that a real-life human venous leg ulcer experiences.

In previous work, we reported on the statistical reliability of standard early wound healing rate measurements for predicting ultimate closure [13]. While there is significant clinical value for the use of linear healing rates and wound trajectories, neither method is an approximation to a standard mathematical function [14,15]. Regression fitting of data points to a function potentially solves this issue by estimating values for the healing rate constant (or contraction constant) independent of initial wound size and geometry. Prediction intervals, goodness-of-fit statistics, and residual error plots may lend more analytical information to the nature of the wound healing process than summary statistics of standard healing rates. Nonlinear curve fitting algorithms, primarily using a Gompertz function, have been applied to serial wound measurements with positive results [16-19]. Regression curve fitting may be a powerful method to mathematically describe contraction and epithelialization rates in a clinical context.

The results of our study strongly suggest that wound surface area reduction for healing VLUs, particularly early in the treatment phase, follows an exponential decay curve. It is inaccurate to claim, though, that wound healing per se follows an exponential decay/growth process. In our study, for example, a few wounds that worsened with time projected with significance to an exponential growth model, which can not be sustained over time due to anatomical limits. It is also not clear whether chronic wounds that heal over a longer time frame (i.e. 6–9 months) follow a similar exponential decay pattern. Further, we have not stratified the data to investigate the influence of patient age, wound duration and various comorbidities and medications on adherence to the model.

There is also additional debate as to whether wound surface area is the best metric for wound size quantification. Wounds that present with significant depth are not adequately quantified by the 2-dimensional surface area measurements used in our regression tests; this may corrupt some outcome predictions. Similarly, wounds of equivalent surface area but of varying geometrical shapes may be best represented by linear healing parameter, wound perimeter, or perimeter/area ratio measurements.

Another important flaw in the application of a generalized exponential model to wound healing data is that the model only truly fits some contraction and epithelialization mechanics. While many VLUs follow an exponential healing pattern, in general these lesions are relatively superficial and in some wounds contraction may be less important than granulation and epithelialization; in addition the mode of healing may be influenced by various therapeutic modalities. An exponential decay function does not intersect at zero; the limit of the function is zero only as *b *(healing rate) or *x *(time) gets very large in scale. This further supports the notion of a wound contraction rate via an exponential decay model, followed by or partially combined with an unmodeled proliferation rate of the wound margins during epithelialization. Often times it was observed in this study that regression residual variation was negative in trend as the wound reached closure. This suggests that the process of epithelialization during the final stages of wound healing may have not fit the model accurately.

## Conclusion

It is common observation that many VLUs heal with some amount of wound contraction; indeed this is demonstrated by the small size of the final scars that come from much larger initial lesions. The effect of contraction in wound healing helps to explain why the exponential decay function upon which we based our analysis is so highly accurate in terms of correlation significance, low residual error, and predictability for healing wounds. This model returns estimates for wound healing rates from a simple, 2-parameter equation that can be reduced to a one-parameter linear equation if necessary. We believe that the simplicity of this model would enable physicians and nurses to incorporate it in clinical decision making at the bedside. It is also convenient that application of this model does not require mathematical transformation of the actual wound measurements. The ability to quantify healing rates as coefficient estimates from a standard statistical test allows hypothesis testing on treatment efficacy, and may circumvent the well known drawbacks of using a binary endpoint (healed or unhealed) at an arbitrarily selected point in time. However, more research is required to develop a mathematical formula that accurately fits the entire process of wound closure. We recommend that additional clinical researchers use this methodology for analyzing wound healing rates to assess its prognostic ability and its accuracy as a mathematical wound healing model. A validated mathematical model that could reduce the duration of clinical trials by even a couple of months would lower the cost of large clinical trials by millions of dollars and enable useful products with small commercial significance to come to market.

## Competing interests

The study was funded by Advanced Biohealing. Based on the materials studied, the authors declare they have no competing interests.

## Authors' contributions

MC performed the bulk of the analysis with contributions from DEE, DGA and JM. DEE, DGA, TP, KH and MC performed the bulk of the write-up of the manuscript. The authors have read and approve the manuscript.

## Pre-publication history

The pre-publication history for this paper can be accessed here:


